# Association between processing speed and segregation/integration of large-scale functional networks in middle-aged and older people living with HIV

**DOI:** 10.21203/rs.3.rs-7303216/v1

**Published:** 2025-08-27

**Authors:** Monica M. Diaz, Matthew G. Harris, Jacqueline M. Koble, Keely Copperthite, Jordan Jimenez, Eran Dayan

**Affiliations:** University of North Carolina at Chapel Hill School of Medicine; University of North Carolina at Chapel Hill School of Medicine; University of North Carolina at Chapel Hill School of Medicine; University of North Carolina at Chapel Hill School of Medicine; University of North Carolina at Chapel Hill School of Medicine; University of North Carolina at Chapel Hill School of Medicine

**Keywords:** HIV, aging, functional MRI, segregation/integration, processing speed, functional networks

## Abstract

**Background:**

Neurocognitive impairment (NCI) is a common comorbidity among aging people with HIV (PWH), despite effective antiretroviral therapy (ART). Processing speed is often the earliest affected cognitive domain and may be linked to disrupted functional brain network organization. This study investigated whether the balance of segregation and integration in large-scale functional networks is associated with processing speed in middle-aged and older PWH.

**Methods:**

In a prospective, cross-sectional study, 26 virologically suppressed PWH aged ≥ 50 years underwent neuropsychological testing and resting-state functional MRI (rs-fMRI). Functional brain networks were constructed using a 300-node cortico-subcortical parcellation. System segregation index and node-level participation coefficient (PC) were calculated to quantify the global and local balance between integration and segregation, respectively. Associations with age-adjusted Wechsler Adult Intelligence Scale (WAIS-III) Symbol Search (WAISsys) T-scores were assessed using regression and correlational analyses.

**Results:**

Higher system segregation within associative networks was significantly associated with better WAISsys T-scores (β = 0.544, p = 0.004), whereas segregation in sensorimotor networks was not. The majority of nodal PC values were negatively correlated with WAISsys T-scores, indicating that lower processing speed was associated with less segregated and more integrated connectivity. Nodes showing the strongest negative associations with WAISsys T-scores were disproportionately located in the default mode and frontoparietal networks.

**Conclusions:**

In middle-aged and older PWH, greater segregation within associative networks is linked to better processing speed. Disruptions in network segregation and modularity, especially in cognitive control systems, may be associated with processing speed deficits despite viral suppression. These findings highlight the importance of functional brain network topology and organization as a potential biomarker for cognitive aging in HIV.

## Background

Neurocognitive impairment (NCI) in the setting of HIV (HIV-NCI) remains a common comorbidity affecting up to 50% of people with HIV (PWH)(1), despite the use of antiretroviral treatment (ART) to suppress viral replication. HIV-NCI in ART-treated patients is usually characterized by a mild cognitive impairment with impairments in multiple cognitive domains (2). Processing speed, in particular, is among the major cognitive domains impacted in HIV-NCI (3, 4), and poor performance in tests of processing speed in PWH is strongly correlated with health-related quality of life (5). Neuropathologically, slowed processing speed in HAND is thought to result from widespread subcortical and frontostriatal dysfunction, in regions commonly affected by HIV(6, 7), yet the mechanisms that underlie alterations in processing speed remain incompletely understood.

Middle-aged brains undergo structural and functional changes^8^ that may be exacerbated in PWH. The balance between integration and segregation in large-scale brain networks is critical for multiple cognitive domains (9, 10), with domains such as processing speed linked to increased segregation (9). Using mutual connectivity analysis and network based statistics to analyze resting-state functional MRI (rs-fMRI) results in multiple resting state networks, one study found that compared to people without HIV (PWoH), PWH with HIV-NCI had disrupted functional connectivity primarily in the posterior sections of the default mode network (DMN), a region which has been associated with cognitive performance (11). In another study of 98 young-adult PWH and 44 PWoH, PWH displayed aberrant functional integration and segregation even at early stages of NCI (12). This demonstrates the utility of using measures of integration and segregation in large-scale functional networks based upon fMRI to detect brain mechanisms associated with NCI in PWH.

**Figure 1:**
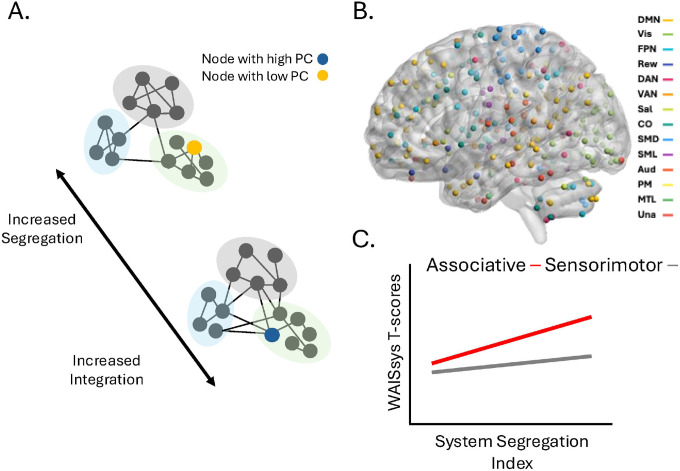
Study outline. (A). The study examined if the balance between integration and segregation in large-scale functional networks relates to WAISsys T-scores in people with HIV (PWH). Global levels of segregation and integration were quantified using the system segregation index, where increased integration reflects stronger connectivity *between* relative to *within* network communities while increased segregation reflects the opposite relationship between the two. Integration and segregation at the level of individual nodes was additionally quantified with the participation coefficient (PC), similarly measuring whether nodes tend to interact mostly within their own network community (low PC), or rather with a more diverse set of network communities (high PC). (B) We considered global and local levels of integration and segregation among large-scale functional networks represented in a whole-brain 300 nodes parcellation. (C). We hypothesize that associations between system segregation index scores and WAISsys T-scores will be more pronounced in associative, rather than sensorimotor networks. Aud: auditory network; CO: cingulo-Opercular network; DAN: dorsal attention network; DMN: default mode network; FPN: frontoparietal network; MTL: medial temporal lobe network; PM: parieto-medial network; Rew: reward network; Sal: salience network; SMD: somatomotor dorsal network; SML: somatomotor lateral network; Una: unassigned; VAN: ventral attention network; Vis: visual network

Therefore, our study aimed to examine whether the extent of segregation/integration within subjects’ large-scale functional networks is linked to processing speed, as assessed using age-adjusted WAISsys scores (henceforth, WAISsysT scores). In our study, we queried each subject’s balance between segregation and integration using the system segregation index (13, 14) (Fig. 1A), a global metric quantifying the ratio between connectivity within and between large-scale functional networks. We additionally quantified the participation coefficient (PC) for each node in the network (Fig. 1A), denoting the diversity of connections incident to each node. As in earlier research (13, 15, 16), we considered the extent of segregation/integration in both associative and sensorimotor brain networks. Namely, we constructed individual-subject brain networks using a 300 node cortico-subcortical brain parcellation, composed of both associative and sensorimotor functional networks (Fig. 1B). We expected the association between network segregation and WAISsysT scores to be more pronounced in associative networks, than in sensorimotor networks (Fig. 1C).

## Methods

### Study Participants

This was a cross-sectional, prospective study of PWH attending the University of North Carolina Infectious Diseases clinic. All participants were aged ≥50 years, on stable ART for ≥1 year and had an undetectable plasma HIV viral load within the past 6 months. We also excluded PWH with a history of confounding neurologic conditions that could impair cognition (i.e. stroke, prior head injury, untreated or undertreated psychotic disorder, active alcohol or substance use disorder, opportunistic CNS infection, active or untreated Hepatitis C infection, autoimmune disorder requiring immunotherapy, active malignancy, liver cirrhosis). We additionally excluded participants who had a relative contraindication to undergoing an MRI including: history of foreign metal object, pacemaker, or other foreign device in the body that is not MRI compatible, history of moderate to severe claustrophobia, history of prior intolerance to MRIs due to reasons other than claustrophobia (i.e. inability to lie flat) or were currently pregnant.

### Study Procedures

All participants were screened for eligibility prior to study enrollment and provided written informed consent prior to enrolling in the study. All participants underwent a medical evaluation including a neuromedical questionnaire regarding the patient’s past medical and psychiatric history obtained from the patient and then corroborated by reviewing the patient’s medical chart.

### Neuropsychological Testing

All participants underwent a battery of neuropsychological tests administered by a research assistant who had been trained in administering a neuropsychological test battery, supervised by a neuropsychologist on the team (MH). The test battery was comprised of a series of neuropsychological tests assessing five cognitive domains previously used in HIV neurological trials(17) and aligning with AIDS Clinical Trial Group neurocognitive studies(18). These measures have published norms used in other ACTG studies(19) that were utilized. The tests and corresponding cognitive domains assessed included: Executive Function (Stroop Interference(20), Trail Making B(21)); Motor (Grooved pegboard(21)); Learning and memory (Hopkins Verbal Learning(23), Brief Visual Memory(24)); Processing speed (Trail Making A(21), WAIS-III Symbol Search(22), WAIS-III Coding(22); and Verbal (Semantic Verbal Fluency [COWA, Animals], Letter Fluency [FAS](25)), along with an estimate of baseline intellectual ability (WRAT-4 Reading). Here, our focus was on age-adjusted WAIS-III Symbol Search (WAISsys T-scores), as a measure of processing speed. We selected the WAISsys as it has the least motor involvement (Coding and Trails A involve require more motor control) so it is a measure of non-motor processing.

### MRI Data Acquisition and Preprocessing

Multimodal MRI data were acquired on a Siemens 3T Prisma scanner. Structural 3D T1-weighted Magnetization Prepared RApid Gradient Echo (MPRAGE) images using GRAPPA were acquired with the following acquisition parameters: TR = 2300ms, TE = 2.98ms, Slice thickness = 1mm, flip angle = 9°. Functional T2*-weighted images were acquired with an Echo Planar Imaging (EPI) sequence using the following parameters: TR = 800ms, TE = 37ms, Slice thickness = 2mm, interleaved acquisition, 737 volumes.

Preprocessing of MRI data was carried out using the Conn toolbox (Conn22.a) and SPM12, both running on MATLAB (R2023a). Structural images were first segmented into gray matter, white matter, and cerebrospinal fluid (CSF). Functional images underwent realignment and unwarping, slice-timing correction, co-registration to structural images, spatial normalization, and motion estimation and outlier detection. White matter, CSF, and 12 motion parameters (6 motion parameters and their first order derivatives) were included as nuisance regressors. Outlier volumes with movement exceeding 0.9 mm or a Z-score greater than 5 were regressed out. The data were then detrended and a temporal band-pass filter was applied to filter out Blood Oxygenation Level Dependent (BOLD) signal frequencies outside the 0.008–0.09 Hz range.

### Network construction and analysis

Preprocessed BOLD time-series data were used for network construction. Connectivity matrices were constructed for each subject using a whole-brain cortico-subcortical parcellation comprised of 300 regions of interest (ROIs)/nodes(26). Edges in these connectivity matrices represented the strength of correlation between each pair of ROIs/nodes (Fisher-Z transformed), resulting in a 300 × 300 unweighted connectivity matrix for each individual subject. We then calculated, for each subject, the system segregation index(13, 14), defined as:

1 $$\frac{\bar{Z_{w}}-\bar{Z_{b}}}{\bar{Z_{w}}},$$

where $\bar{Z_{w}}$ denotes mean connectivity (correlation) strength *within* networks, whereas $\bar{Z_{b}}$ denotes mean connectivity *between* networks. This analysis further differentiated between the system segregation index within associative (CinguloOpercular, default mode, dorsal attention, frontoparietal, salience and ventral attention) and sensorimotor (auditory, somatomotor dorsal, somatomotor lateral and visual) networks. In our calculations of the system segregation index, negative edges were replaced with 0, but otherwise edges were fully weighted.

Next, to derive insights on more local, node-level, patterns of segregation and integration we calculated the participation coefficient (PC)(27) for each node in the 300 node parcellation. This metric reflects the extent of diversity in nodal connections within networks. Thus, for every node *i*, and network community *m* :

2 $$PC_{i}={\sum_{m=1}^{M}\left[\frac{K_{i}(m)}{K_{i}}\right]}^{2},$$

where *K*_*i*_ *(m)* represents connections of node *i* with nodes within network community *m*, while *K*_*i*_ represents the connections of node *i* over all network communities in the parcellation. Higher PCs are thus given to nodes with diverse sets of connections (i.e., connections that extend beyond a given node’s network community), while lower PCs are given to nodes whose connectivity is more restricted. In the analysis of PC values matrices were binarized at 15 densities ranging from 10–25% of edges, in steps of 1%, applied at the individual subject level. Results were then averaged across all densities to derive single measures of PC for each node and each subject.

### Statistical Analyses

Associations between the system segregation index serving as a covariate of interest and WAISsysT score serving as the dependent variable were calculated via regression analysis, adjusting for sex. Standardized regression coefficients are reported. Correlations between nodal PC values and WAISsys T-scores were calculated using Pearson’s correlation. The community affiliation of nodes whose PC values showed the strongest correlations with WAISsys T-score was compared to the general community affiliation of network communities in the parcellation using the Chi-squared test. Statistical analyses were carried out with JASP 0.16.4 or MATLAB (R2023a).

## Results

We analyzed data from 26 PWH with mean (standard deviation [SD]) age 61.5 (6.8) years, 19% female with a mean (SD) educational level of 14.1 (2.4) years). The median (IQR) number of years with HIV was 14 (12, 16), recent absolute CD4 count was 711 (436, 840) and nadir CD4 count was 414 (184, 559).

**Figure 2:**
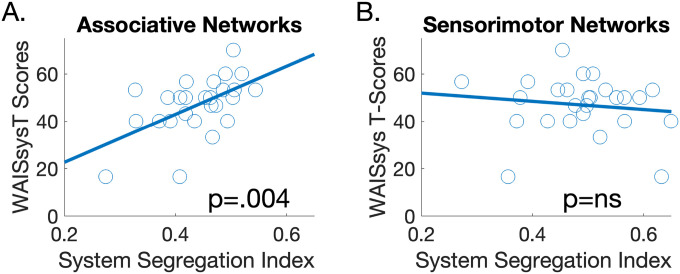
Associations between the System Segregation Index and WAISsys T-scores. (A). Higher segregation in associative networks was associated with better WAISsys T-scores (p=.004). (B.) The association between segregation in sensorimotor networks and WAISsys T-scores was not significant.

We first evaluated the extent to which the balance between integration and segregation in subjects’ functional networks, as measured using the system segregation index(13, 14) was associated with their WAISsys T-scores. We further differentiated between integration/segregation in associative network, where a stronger association was expected given this circuitry’s documented role in cognitive performance, and sensorimotor networks where a weaker association was expected. We found that higher system segregation index scores in associative networks were associated with higher WAISsys T-scores (β = 0.544, p = 0.004. Figure 2A). On the other hand, the association between system segregation index scores in sensorimotor networks and WAISsys T-scores was not significant (β =−0.135, p = 0.38. Figure 2B). These results thus suggest that higher segregation in associative large-scale functional networks is linked to better processing speed in our sample.

**Figure 3:**
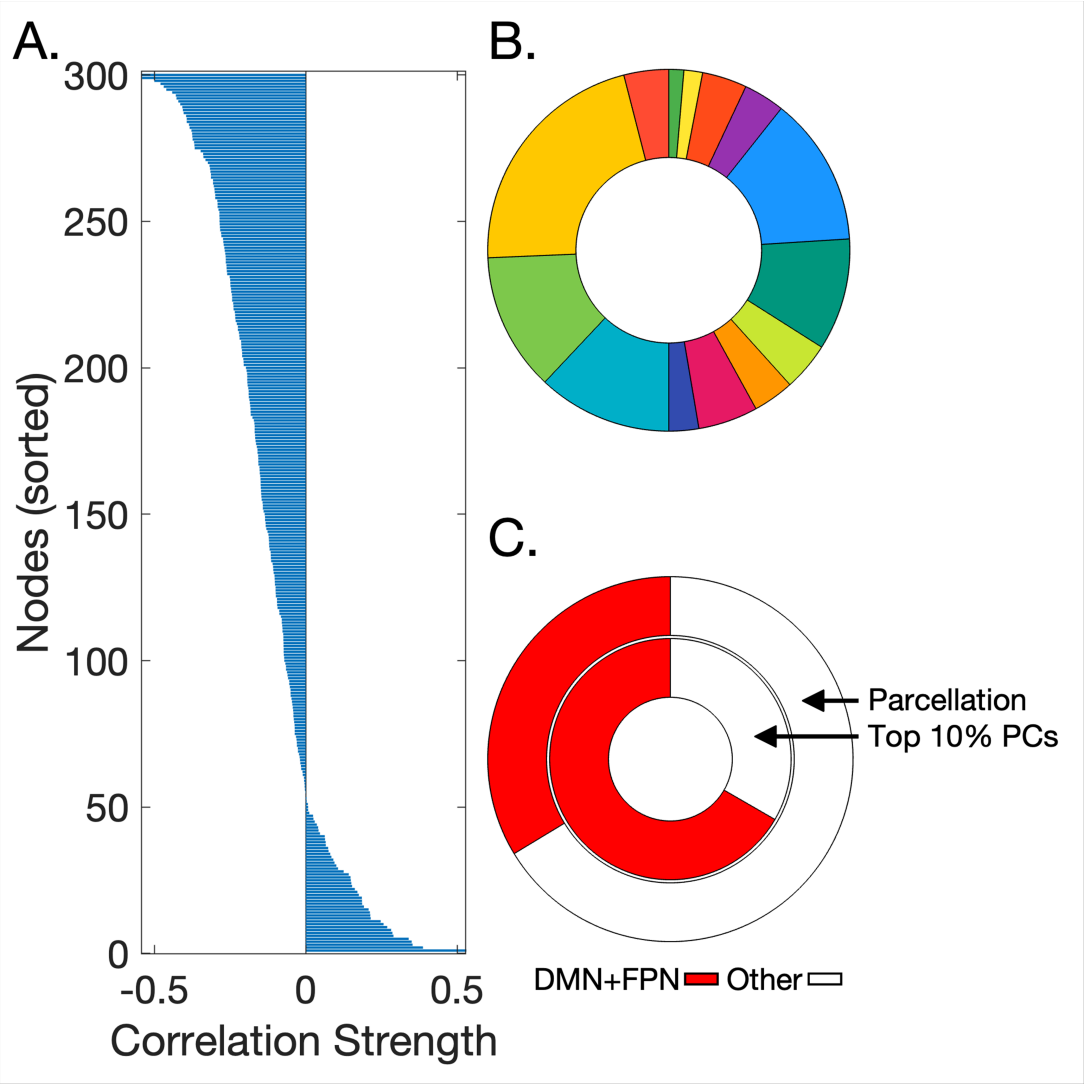
Associations between nodal participation coefficients and WAISsys T-scores. (A). Associations between nodal PC values and WAISsys T-scores are displayed for each node and sorted by correlation strength. 82% of the correlations were negative. (B). The size of each network community within the 300 node parcellation. (C) Proportion of nodes in the DMN and FPN networks, relative to all other networks are shown in relation to the full parcellation (outer ring) and for the nodes whose PC values had the top 10% strongest negatives correlations with WAISsys T-scores (inner ring). All abbreviations are as in Fig. 1.

The results reported above are based on a global measure of integration/segregation in large-scale functional networks. Next, to obtain additional insights on the more local patterns of integration and segregation in the brain as they relate to processing speed, we calculated for each of the 300 nodes in the parcellation, and across all subjects, the PC(27), a nodal measure expressing the extent of diversity in nodal connections within networks. We first correlated each nodal PC value against WAISsys T-scores (Fig. 3A). 82% of the correlation values were negative. That is, for the vast majority of nodes in the parcellation, lower WAISsys T-scores were associated with more integrated and less segregated connectivity.

We next determined the community affiliation of nodes whose PC values showed the strongest correlations with WAISsys T-scores, when considering the size of each network community within the 300 node parcellation (Fig. 3B). We thus considered the nodes whose PC values showed the strongest (top 10%) negative correlations with WAISsys T-scores. Two-thirds of these nodes belonged to either the default mode network (DMN) or the frontoparietal network (FPN). This proportion was significantly different than the proportion of DMN and FPN nodes within the entire parcellation (χ^2^ = 12.79, p < .001) (Fig. 3C), highlighting the centrality of DMN and FPN topology in relation to processing speed in PWH.

## Discussion

Our study evaluated whether the balance of segregation versus integration in large-scale functional brain networks relates to processing speed in middle-aged and older PWH. We used the WAIS-III Symbol Search T-scores as a measure of processing speed and queried global and local measures of integration and segregation in associative versus sensorimotor networks. We found that higher segregation within associative networks was significantly associated with better WAISsys T-scores. We also found that the majority of nodal PC values were negatively correlated with WAISsys T-scores, indicating that lower processing speed was associated with less segregated and more integrated connectivity. Nodes showing the strongest negative associations with WAISsys T-scores were disproportionately located in the DMN and FPN.

We show that higher segregation in associative networks, but not in sensorimotor networks, was associated with better T-scores on the WAISsys test. Lifespan studies have consistently shown that aging results in loss of segregation(13, 28), and related changes in modularity (29–31), which can be throughout as reflecting age-associated dedifferentiation occurring among neuronal systems(32, 33). Intact cognition necessitates a balance between integration and segregation(10). In older adults, decreased segregation in associative networks including the FPN and DMN has been shown to relate to cognitive abilities, including processing speed (32, 34). Adding to these results our findings demonstrate that better processing speed performance in middle-aged and older PWH is associated with increased segregation in associative large-scale functional networks.

Complementing the results on global patterns of integration/segregation, we also observed that lower WAISsys T-scores were associated with more integrated and less segregated local (node-level) patterns of connectivity, measured with the PC metric. Put differently, loss of specialization and connection diversity in individual nodes may impair cognitive performance, particularly processing speed, in middle-aged and older PWH. These findings align with the demonstration that excessive integration may diminish system specificity, reducing processing speed and cognitive efficiency(35). Altogether, our results suggest that both local and global patterns of integration and segregation in associative brain networks underlie intact (and deficient) processing speed in middle-aged and older PWH.

Our findings underscore the centrality of DMN and FPN topology in relation to processing speed in PWH. The topological properties of these two networks have been consistently linked to cognitive performance in aging and age associated neurogenerative diseases. Namely, the topological robustness of the DMN and FPN is associated with cognitive performance in middle-aged and older healthy adults(36), with the latter being specifically predictive of cognitive decline in Parkinson’s disease (37). The FPN (38–40) and DMN (40–42) are particularly vulnerable to the effect of aging, and their reserve properties (i.e., redundancy in their connectivity patterns(28, 43, 44)) were shown to counter age-associated changes in their topology to minimize cognitive decline(45). Our findings reveal that the topological properties of the DMN and FPN also play a role in processing speed performance among middle-aged and older PWH.

Our study has limitations. Although the study has a small sample size, we report one of the first findings to demonstrate relationships between the integration and segregation of networks and processing speed in middle-aged and older PWH. This was a cross-sectional study, so longitudinal studies would be needed to determine if integration/segregation changes occur over time as PWH age. We did not measure the association with HIV characteristics in this particular study, limiting generalizability. However, all participants had an undetectable viral load and an absolute CD4 count of 200. Finally, we did not include a control group in our study, so insights into disease-specific mechanisms will have to be established in future research.

## Conclusions

In middle-aged and older PWH, greater segregation within associative networks is linked to better processing speed performance. Disruptions in network segregation, especially in cognitive control systems, may be associated with processing speed deficits despite viral suppression. These findings highlight the importance of functional brain network organization and topology as a potential biomarker for cognitive aging in HIV. Future work may determine longitudinal changes and disease-specific mechanisms in these brain systems as they relate to processing speed and other cognitive domains impacted by NCI in PWH.

## Data Availability

All data will be made available with request to the corresponding authors.

## Abbreviations

NCI: Neurocognitive impairment
ART: antiretroviral therapy
BOLD: Blood Oxygenation Level Dependent
CSF: cerebrospinal fluid
DMN: default mode network
EPI: Echo Planar Imaging
fMRI: functional MRI
FPN: frontoparietal network
IQR: interquartile range
MPRAGE: Magnetization Prepared RApid Gradient Echo
PC: participation coefficient
PWH: people with HIV
ROIs: Regions of Interest
rs-fMRI: resting-state functional MRI
SD: standard deviation
WAIS: Wechsler Adult Intelligence Scale
WAISsys: WAIS-III Symbol Search
